# Associations of Bcl-2 rs956572 genotype groups in the structural covariance network in early-stage Alzheimer’s disease

**DOI:** 10.1186/s13195-018-0344-4

**Published:** 2018-02-08

**Authors:** Chiung-Chih Chang, Ya-Ting Chang, Chi-Wei Huang, Shih-Jen Tsai, Shih-Wei Hsu, Shu-Hua Huang, Chen-Chang Lee, Wen-Neng Chang, Chun-Chung Lui, Chia-Yi Lien

**Affiliations:** 1 Department of Neurology, Cognition and Aging Center, Kaohsiung Chang Gung Memorial Hospital, Chang Gung University College of Medicine, No. 123 Ta-Pei Road, Niaosung, Kaohsiung County 833 Taiwan; 2 Psychiatric Department, Taipei Veterans General Hospital, Taipei, Taiwan; 3 Psychiatric Division, School of Medicine, National Yang-Ming University, Taipei, Taiwan; 4 Department of Radiology, Kaohsiung Chang Gung Memorial Hospital, Chang Gung University College of Medicine, Kaohsiung, Taiwan; 5 Department of Nuclear Medicine, Kaohsiung Chang Gung Memorial Hospital, Chang Gung University College of Medicine, Kaohsiung, Taiwan; 6 Division of Medical Imaging, E-Da Cancer Hospital and I-Shou University, Kaohsiung, Taiwan

**Keywords:** Anatomical structural covariance, Default mode network, Posterior cingulate cortex, Executive control network, Bcl-2, Polymorphism

## Abstract

**Background:**

Alzheimer’s disease (AD) is a complex neurodegenerative disease, and genetic differences may mediate neuronal degeneration. In humans, a single-nucleotide polymorphism in the B-cell chronic lymphocytic leukemia/lymphoma-2 (Bcl-2) gene, rs956572, has been found to significantly modulate Bcl-2 protein expression in the brain. The Bcl-2 AA genotype has been associated with reduced Bcl-2 levels and lower gray matter volume in healthy populations. We hypothesized that different Bcl-2 genotype groups may modulate large-scale brain networks that determine neurobehavioral test scores.

**Methods:**

Gray matter structural covariance networks (SCNs) were constructed in 104 patients with AD using T1-weighted magnetic resonance imaging with seed-based correlation analysis. The patients were stratified into two genotype groups on the basis of Bcl-2 expression (G carriers, *n* = 76; A homozygotes, *n* = 28). Four SCNs characteristic of AD were constructed from seeds in the default mode network, salience network, and executive control network, and cognitive test scores served as the major outcome factor.

**Results:**

For the G carriers, influences of the SCNs were observed mostly in the default mode network, of which the peak clusters anchored by the posterior cingulate cortex seed determined the cognitive test scores. In contrast, genetic influences in the A homozygotes were found mainly in the executive control network, and both the dorsolateral prefrontal cortex seed and the interconnected peak clusters were correlated with the clinical scores. Despite a small number of cases, the A homozygotes showed greater covariance strength than the G carriers among all four SCNs.

**Conclusions:**

Our results suggest that the Bcl-2 rs956572 polymorphism is associated with different strengths of structural covariance in AD that determine clinical outcomes. The greater covariance strength in the four SCNs shown in the A homozygotes suggests that different Bcl-2 polymorphisms play different modulatory roles.

**Electronic supplementary material:**

The online version of this article (10.1186/s13195-018-0344-4) contains supplementary material, which is available to authorized users.

## Background

In Alzheimer’s disease (AD), the self-aggregation of amyloid fibrils and accumulation of amyloid plaques represent an important early pathological finding. Whereas the presence of amyloid may trigger downstream network degeneration, the presence of the antiapoptotic protein, B-cell chronic lymphocytic leukemia/lymphoma-2 (Bcl-2), may offer protection by regulating cellular resilience and apoptosis pathways [1]. In the mammalian central nervous system, increased expression of Bcl-2 protein has been shown to promote axon regeneration [2]. In patients with AD, the overexpression of Bcl-2 protein in surviving glia surrounding amyloid plaques suggests its role against neuroinflammation [3]. In addition, nicergoline, a drug that upregulates Bcl-2 protein expression and acts against β-amyloid cascades, has been used clinically to treat age-dependent cognitive impairment [4].

In humans, a single-nucleotide polymorphism in the Bcl-2 gene, rs956572, has been found to significantly modulate protein and messenger RNA (mRNA) expression levels [5, 6]. The Bcl-2 AA genotype group has been associated with reduced Bcl-2 levels [6]. In bipolar disorders, lower Bcl-2 protein and mRNA levels have been reported in the frontal cortex [7]. Subsequent reports in healthy elderly subjects have suggested that genetic variations in Bcl-2 modulate intracerebral structures, including the hippocampus, ventral striatum, and precuneus [8, 9]. The AA variant has also been associated with lower gray matter (GM) volume in healthy individuals [8]. However, the genetic associations of Bcl-2 on network influences remain to be explored in patients with AD.

Recently, resting-state or task-based functional magnetic resonance imaging has been used to map the network connectivity. With subjects in a resting state [10], spontaneously correlated low-frequency signal fluctuations occurring within spatially distinct, functionally related cortical-subcortical regions can be measured. One functional network in a task-free state in healthy subjects is referred to as the *default mode network* (DMN), which includes the posterior cingulate cortex (PCC), anterior medial prefrontal cortex, medial temporal lobe, lateral temporal cortex, and inferior parietal lobule. The DMN is regarded to be an early neuroimaging biosignature [11], and a recent report suggested that the DMN may be comprised of multiple spatially dissociated but interactive components [12], of which two subsystems are of particular interest. The “medial temporal lobe subsystem,” anchored by the entorhinal cortex and the hippocampus, includes the ventral medial prefrontal cortex, posterior inferior parietal lobule, retrosplenial cortex, parahippocampal cortex, and hippocampal formation. This subsystem is involved in mental scene construction and decision making based on retrieval of memory. The “dorsal medial prefrontal cortex subsystem” (or the midline core subsystem) plays a role in self-relevant and affective decision making. It includes the core of the PCC and anterior medial prefrontal cortex, and the cortical hubs of the temporoparietal junction and the lateral temporal and temporal poles.

The salience network is anchored by the frontoinsular cortex and dorsal anterior cingulate cortex [13]. Enhanced resting-state functional connectivity of the salience network has been reported in patients with AD [14], and possible mechanisms have been hypothesized to reflect a compensatory mechanism for the weakened posterior hubs [15]. The executive control network (ECN) represents another network that shows increased functional connectivity in patients with AD [16, 17]. Anchored by the dorsolateral prefrontal cortex (DLPFC), the ECN plays a role in functions such as sustained attention, working memory, and response selection and suppression [13].

Recent research suggests that regions which are highly related show covariance in morphometric characteristics, so-called structural covariance. It has also been shown that structural covariance patterns are associated with structural or functional connectivity [18]. In structural covariance pattern analysis, factors such as genetic variations and developmental, degenerative, and disease staging are important covariates of interest [18]. Structural covariance networks (SCNs) can be used to investigate spatial associations of Bcl-2 genotype groups in AD.

In this study, we investigated whether the SCN in AD may represent an endophenotype of the Bcl-2 rs956572 genetic polymorphism. Spreng and Turner reported that the Bcl-2 AA genotype may be a risk factor for neuronal apoptosis and oxidative stress [19]. Therefore, we stratified our patients according to the Bcl-2 rs956572 genotype as Bcl-2 G carriers (*n* = 76) and A homozygotes (*n* = 28), matched for confounding factors including sex, age, education level, apolipoprotein E4 status, duration of disease, and Mini Mental State Examination (MMSE) score. Because the Bcl-2 genotypes have been reported to influence psychiatric presentation in bipolar disorders [5, 6, 20, 21], we also included Neuropsychiatric Inventory (NPI) scores to evaluate whether the genotype groups affected the behavioral presentations.

## Methods

This study was conducted in accordance with the Declaration of Helsinki and was approved by the Institutional Review Board of Chang Gung Memorial Hospital. Both the patients and their caregivers provided written informed consent. The study participants were treated at the Cognition and Aging Center, Department of General Neurology, Kaohsiung Chang Gung Memorial Hospital (male/female ratio, 51/53). Subjects were included on the basis of a consensus of panels composed of neurologists, neuropsychologists, neuroradiologists, and experts in nuclear medicine [22]. AD was diagnosed according to the International Working Group criteria [23] for a clinical diagnosis of typical AD. The Clinical Dementia Rating scores were 0.5 or 1. All of the patients with AD were under stable treatment with acetylcholinesterase inhibitors from the time of diagnosis. The exclusion criteria were a history of clinical stroke, a modified Hachinski ischemic score > 4 [24] and depression.

### Clinical assessment

After enrollment, demographic data and family history were recorded, and physical and neurological examinations were performed. General cognitive function was assessed using the MMSE [25]. Verbal and nonverbal episodic memory were assessed using a Chinese version of the Verbal Learning Test [26] and the Rey-Osterrieth Complex Figure Test after a 10-minute delay [27]. Language screening was performed using the 16-item Boston Naming Test [28], a three-step comprehension test, and a semantic verbal fluency test. Visuospatial abilities were assessed using a modified Rey-Osterrieth Complex Figure Test and the number-location test from the Visual Object and Space Perception Battery [29]. Frontal lobe function was assessed using digit-forward and backward-span, design fluency, Stroop interference, and modified Trail Making Test B tests [30]. For the behavioral observations, we used the 12-item version of the NPI [31], with scores ranging from 0 to 144.

### Genotyping

Genomic DNA was extracted from blood using a commercial kit (Gentra Puregene Blood Kit; Qiagen, Hilden, Germany), followed by the genotyping procedures for rs956572 using the PCR restriction fragment length polymorphism method [8, 9] The ancestral allele G yielded three bands of 298, 108, and 161 bp, whereas the mutant allele A yielded two bands of 406 and 161 bp. All of the participants’ scores are summarized according to genotype group (G carriers and A homozygotes) in Table 1.

**Table 1:** Demographic characteristics and neuropsychiatric tests between the Bcl-2 A homozygotes and G carriers in Alzheimer’s disease

Group	G carriers (*n* = 76)	AA (*n* = 28)	*p* Value
Age, years	73.4 (7.6)	71.8 (7.4)	0.32
Education, years	5.7 (5.4)	5.4 (5.1)	0.4
Sex, male/female	42/34	11/17	0.186
ApoE ε4 allele-positive cases, *n* (%)	29 (38.5%)	13 (46.42%)	0.503
Use of rivastigmine/donepezil	37/39	17/11	0.377
Mini Mental State Examination score	21.6 (5.7)	20.6 (5.1)	0.286
CVVLT verbal memory (9)			
Trials 1–4 total	19.6 (7.08)	18.0 (6.9)	0.173
30-second free recall	4.78 (2.6)	4.18 (2.73)	0.265
10-minute free recall	3.52 (3.3)	3.21(2.8)	0.935
Visual memory			
Modified Rey-Osterrieth recall (17)	4.7 (5.2)	4.04 (4.16)	0.67
Visuospatial function			
Modified Rey-Osterrieth copy (17)	14.3 (5.0)	14.9 (4.6)	0.896
Visual Object and Space Perception Battery (10)	6.4 (3.16)	5.8 (3.45)	0.446
Speech and language ability			
Semantic fluency: animal (1 minute)	11.2 (4.63)	10.33 (5.74)	0.387
Boston Naming Test (15)	12.8 (3.0)	11.6 (2.8)	0.04^a^
Comprehension (4)	2.8 (0.9)	2.3 (1.2)	0.053
Executive function			
Digit backward	3.2 (1.3)	3.2 (1.1)	0.758
Stroop interference correct (1 minute)	25.9 (13.1)	21.6 (15.7)	0.178
Design fluency	4.31 (2.8)	4.5 (3.8)	0.612
Trail Making Test time (<120 seconds)	99.2 (32.3)	93.0 (37.9)	0.446
Correct line in Trail Making Test (14)	7.1 (5.4)	7.6 (5.6)	0.742

*CVVLT* Chinese Version Verbal Learning Test

Data are presented as mean (SD); numbers in parentheses following task name are maximal scores

^a^*p* < 0.05, Mann-Whitney *U* test

### Image acquisition

Magnetic resonance images were acquired using a 3.0-T magnetic resonance imaging (MRI) scanner (Excite; GE Medical Systems, Milwaukee, WI, USA). Structural images were acquired for structural covariance analysis using the following protocols: a T1-weighted, inversion recovery-prepared, three-dimensional, gradient-recalled acquisition in a steady-state sequence with repetition time/echo time/inversion time of 8600 milliseconds/minimal/450 milliseconds, a 256 × 256-mm field of view, and a 1-mm slice sagittal thickness with a resolution of 0.5 × 0.5 × 1 mm^3^.

### Data analysis

Image preprocessing and statistical analysis were performed using SPM12 statistical parametric mapping (http://www.fil.ion.ucl.ac.uk/spm/; Wellcome Trust Centre of Cognitive Neurology, University College London, London, UK). The T1-weighted images were reoriented, realigned, and normalized with the standard Montreal Neurological Institute space. The images were then segmented into GM and white matter. Affine-registered tissue segments were used to create a custom template using diffeomorphic anatomical registration using the exponentiated lie algebra approach. This approach represents one of the highest-ranking registration methods in patients with AD [32]. The modulated and warped images were then smoothed with a Gaussian kernel of 8 mm FWHM.

### SCN analysis

To investigate the SCNs, regional GM volumes of 4 regions of interest (ROIs) were extracted from the 104 preprocessed images. The seed ROI included the right entorhinal cortex (coordinates 25, −9, −28), left PCC (coordinates −2, −36, 35), right frontoinsular cortex (coordinates 38, 26, −10), and right DLPFC (coordinates 44, 36, 20). The main purpose of this study was to delineate the topography of SCN in terms of Bcl-2 functional polymorphism rather than to report the SCN differences constructed from the right or left seed. Because pathology or functional connectivity in typical AD is also distributed symmetrically, we did not perform a contralateral seed analysis in this study. According to the literature, these seed regions anchor the DMN medial temporal subsystem (right entorhinal cortex) [33], DMN midline core subsystem (left PCC) [19, 34], salience network (right frontoinsular cortex), and ECN (right DLPFC) [13].

From the modified GM images, the GM volumes of a 4-mm radius sphere around the seed ROI coordinates were calculated, followed by four separate correlation analyses using the extracted GM volumes as the covariates of interest. The patients with AD, G carriers, and A homozygotes were modeled separately. During the construction of the SCNs, the seed volumes were entered as independent variables, and age, MMSE scores, and total intracranial volumes were entered as covariates to control for confounding from the aging process and disease severity. For each group, specific contrasts were set to identify, for each seed ROI, voxels that showed positive correlations within each group (AA and G carriers). The results reflected the SCNs of each group and were thresholded at *p* < 0.05, corrected for a false discovery rate (FDR). Considering the physiological meanings of the SCN clusters and the disproportionate number of cases in two genotype groups, we reported only the significant clusters showing a cluster size > 100 voxels (337.5 mm^3^). Because the experiment was exploratory, the threshold cutoff was set to avoid reporting clusters showing a spatial extent smaller than the predefined size. Meanwhile, to understand whether the distributions of SCN were driven by the number of cases, we also performed SCN in three genotype groups for qualitative comparisons.

Furthermore, to understand how genetic variance may interfere with the structural covariance patterns, voxels showing significant differences in the regression slopes in each ROI were compared, pointing to possible interactions between AA < G carriers or AA > G carriers. The threshold for the resulting statistical parametric maps was thresholded at *p* < 0.05 (FDR-corrected) with a cluster size > 100 voxels.

For the clusters showing significant between-group interactions, a 4-mm radius sphere was placed on the peak voxel, and the GM densities were extracted for regression analysis. To explore the clinical significance of the seed volume and the identified peak voxel volume, we used linear regression analysis to test the relationships with the neurobehavioral scores. The cognitive test scores served as the dependent variable. The threshold was set at *p* < 0.05 with multiple corrections.

### Statistical analysis

Clinical and laboratory data were expressed as mean ± SD. The Mann-Whitney *U* test was used to compare levels of continuous variables of the G carriers and A homozygotes. Spearman’s correlation analysis adjusted for possible confounders was performed to assess associations between continuous variables. The interactions between two genotype groups in covariance strength and their 95% CIs were explored. All statistical analyses were conducted using IBM SPSS Statistics version 20 for Windows® software (IBM, Armonk, NY, USA). Statistical significance was set at *p* < 0.05.

## Results

### Demographic data, cognitive data, and NPI

The demographic and neurobehavioral characteristics of the Bcl-2 A homozygotes and G carriers are shown in Table 1. The genotype distribution did not violate the Hardy-Weinberg principle (chi-square value = 1.65; *p* = 0.19). The G carriers had significantly higher scores in the Boston Naming Test, but the effects were not related to the categories of acetylcholinesterase inhibitors. The total NPI score in the A homozygotes (3.4 ± 5.1) was significantly lower than that in the G carriers (7.5 ± 9.4; *p* = 0.006). The significance was related to the subdomains of aggression (A homozygotes 0.04 ± 0.19; G carriers 0.5 ± 2.1; *p* = 0.04) and sleep (A homozygotes 0.8 ± 2.6; G carriers 2.7 ± 4.6; *p* = 0.009).

### SCN patterns in the two genetic variants

**Fig. 1:**
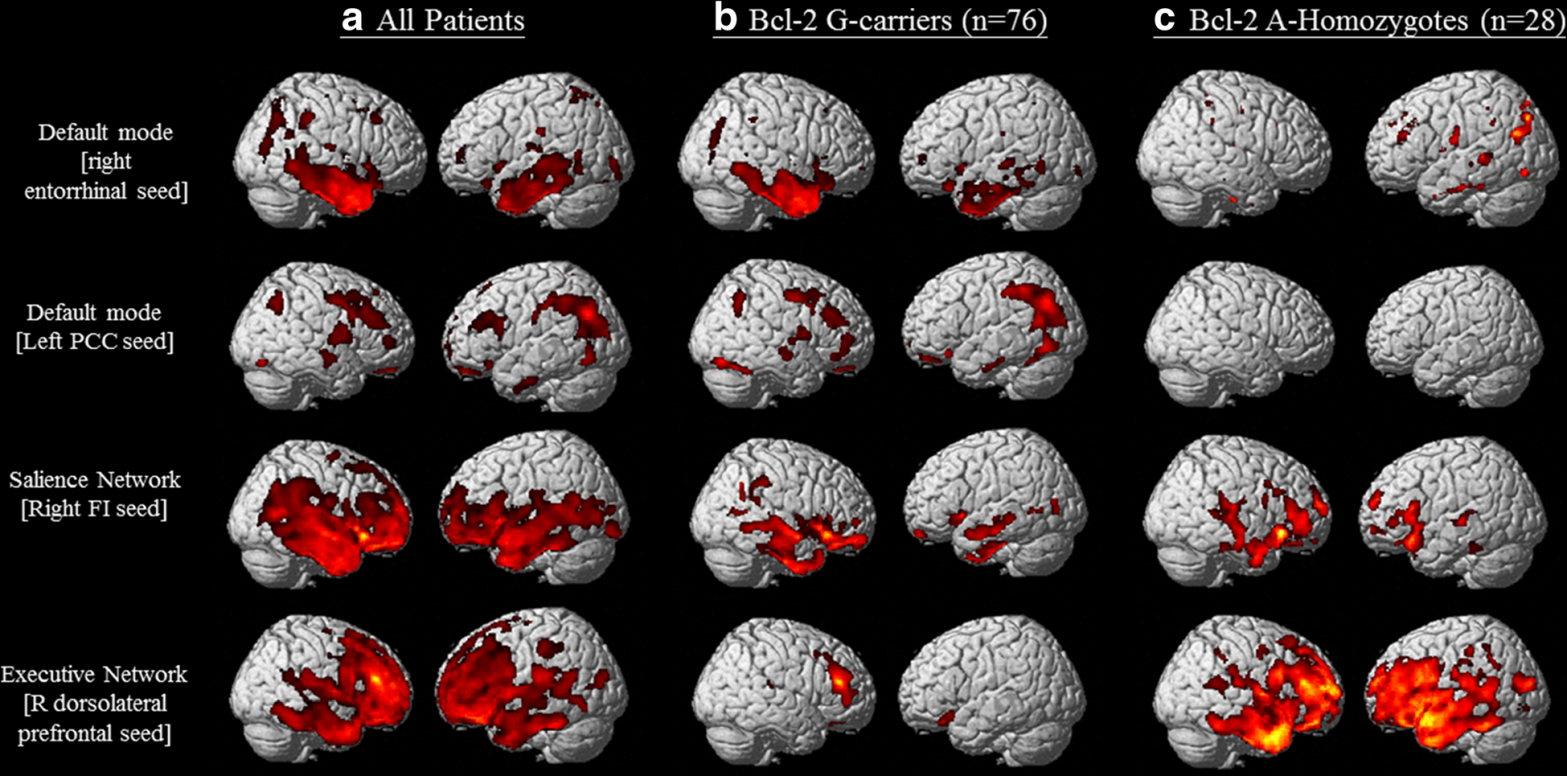
Statistical maps depict brain areas in which the gray matter intensity covaried with four target seeds for separate networks in (**a**) all patients with Alzheimer’s disease (*n* = 104), (**b**) G carriers (*n* = 76), and (**c**) A homozygotes (*n* = 28). Z-statistic maps (*p* < 0.05, corrected with false discovery rate with extended cluster voxels > 100). The images are displayed on a standard brain render. *R* Right; *PCC* Posterior cingulate cortex; *FI* Frontoinsular; *Bcl-2* B-cell chronic lymphocytic leukemia/lymphoma-2 gene (rs956572)

In all of the included patients, regions showing structural associations with the seed regions of each SCN were generally consistent with those reported in the literature [12, 13, 35] (Fig. 1a, Additional file 1: Tables S1–S4). A direct comparison using voxel-based morphometry [36] between the G carriers and A homozygotes showed no significant difference in GM volume with the threshold set at *p* < 0.05, corrected for an FDR with a cluster size > 100 voxels.

The SCN patterns in the G carriers and A homozygotes are presented in Fig. 1b and c and Additional file 1: Table S5–S12. Interestingly, in the ECN, the A homozygotes had a considerably greater extent of structural covariance (number of voxels = 80,476) than the G carriers (number of voxels = 5673), although there were fewer cases in the A homozygote group. The SCNs of the GG, GA, and AA genotype groups are shown in Additional file 2: Figure S1.

### Correlation analysis between seed volume and neurobehavioral scores in the two genotype groups

Although the seed volumes were not significantly different between the G carriers and A homozygotes, correlation analysis suggested that the seed volumes played different roles in the two groups (Table 2). For the G carriers, more cognitive domains showed significant correlations with the test scores in the entorhinal and frontoinsular seeds, whereas most of the cognitive tests were related to the volume of the DLPFC seed in the A homozygotes. For NPI scores, the clinical correlations with the seed volumes did not reach statistical significance.

**Table 2:** Correlation coefficients between seed volume and cognitive test scores

Seed region	R Entorhinal		L Posterior Cingulate		R Frontoinsular		R Dorsolateral prefrontal	
MNI Coordinates	(25,-9,-28)		(-2,-36, 35)		(38, 26,-10)		(44, 36, 20)	
Bcl-2 Genotypes	G-carriers	AA	G-carriers	AA	G-carriers	AA	G-carriers	AA
Seed Volume	0.782	0.786	0.642	0.671	0.647	0.645	0.474	0.447
MMSE	0.220	*0.458* ^b^	*0.273* ^a^	0.191	0.135	0.123	-0.056	*0.571* ^b^
T1 to T4 trial scores	0.216	0.298	0.211	0.123	0.133	0.248	0.124	*0.559* ^b^
30 second recalls	*0.323* ^b^	0.204	0.204	0.278	0.180	0.296	0.073	*0.689* ^b^
10 minute recalls	*0.313* ^b^	0.213	0.193	0.100	0.087	0.123	-0.038	*0.484* ^b^
Modified Rey-Osterrieth Recall	0.179	*0.415* ^a^	0.188	0.120	0.168	0.243	-0.034	*0.478* ^a^
Modified Rey-Osterrieth Copy	-0.006	0.192	0.136	0.081	-0.009	0.271	-0.123	*0.505* ^b^
Visual object and Space Perception	-0.037	-0.006	0.058	-0.087	0.096	-0.204	-0.093	0.144
Semantic fluency: Animal	*0.298* ^a^	-0.002	0.101	0.141	0.167	0.041	0.068	*0.527* ^b^
Boston Naming Test	0.114	0.016	0.149	0.209	0.060	0.170	0.106	*0.459* ^a^
Comprehension	0.079	-0.106	0.253	0.179	0.126	0.106	0.234	0.342
Digit backward	-0.013	-0.018	0.139	0.164	-0.048	0.171	-0.090	0.227
Stroop Interference Correct	*0.381* ^b^	0.259	0.209	0.363	*0.398* ^b^	-0.009	*0.298* ^a^	*0.600* ^b^
Design fluency	*0.26*5 ^a^	0.201	0.025	0.007	0.117	*0.495* ^a^	-0.079	*0.618* ^b^
Trail making test time	-0.162	-0.289	-0.168	-0.169	-0.048	-0.116	0.025	-*0.516* ^b^
Correct line in Trail making	0.192	0.062	0.227	-0.023	*0.261* ^a^	0.160	-0.047	*0.497* ^b^
Neuropsychiatric Inventory	-0.023	-0.037	-0.103	-0.253	0.008	-0.117	-0.047	0.063

Numbers indicate Spearman correlation coefficients, ^a^*p* < 0.05; ^b^ *p* <0.01

*MMSE* Mini-Mental State Examination, *MNI* Montreal Neurological Institute, *R* right, *L* Left

### Analysis of interactions in covariance strength

**Fig. 2:**
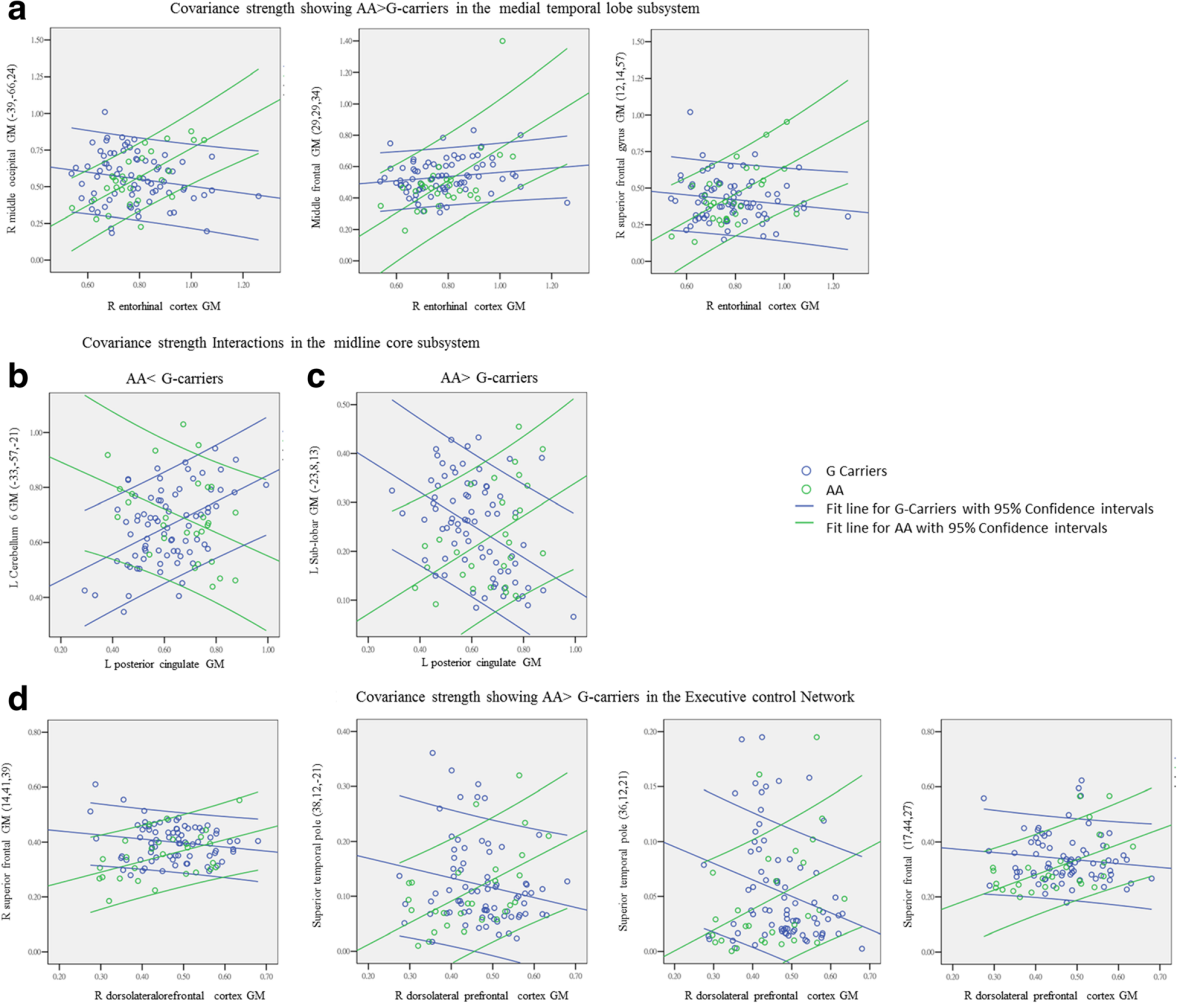
Peak clusters showing significant interactions of B-cell chronic lymphocytic leukemia/lymphoma-2 (Bcl-2) AA and G carriers from the (**a**) default mode network anchored by entorhinal cortex (AA > G carriers); midline core subsystems with interactions of (**b**) AA < G carriers or (**c**) AA > G carriers; (**d**) executive control network by the dorsolateral prefrontal cortex (AA > G carriers). (x, y, z) = Montreal Neurological Institute coordinates. G carriers: *blue*; AA genotype: *green*. The *colored lines* represent the covariance strengths between seed and peak cluster with 95% CIs as boundaries. *GM* Gray matter, *R* right, *L* Left

We further explored interactions of the Bcl-2 genotypes with regard to differences in covariance strength. The relationships are shown in Fig. 2 and Table 3. Within the SCNs anchored to the right entorhinal seed, significant and increased structural covariance was observed in the A homozygotes compared with the G carriers (Table 3, Fig. 2a). The peak clusters showing interactions included the midoccipital and midfrontal regions as well as superior frontal clusters.

**Table 3:** Interactions of Bcl-2 genotypes with pre-defined seed

Seed	Peak regions		Stereotaxic coordinates			Extent	Max T	*P*-value
		Side	x	y	z			
Right entorhinal seed (Medial Temporal Subsystem of DMN)								
G- carriers < AA	Mid-occipital	R	39	-66	24	288	4.34	*<0.0001*
	Mid-Frontal	R	29	29	34	869	3.98	*<0.0001*
	Superior Frontal	R	12	14	57	255	3.97	*<0.0001*
Left posterior cingulate seed (Medline Subsystem of DMN)								
G-carriers > AA	Cerebellum area6	L	-33	-57	-21	928	4.41	*<0.0001*
	Cerebellum area6	L	-23	-55	-20	1137	4.32	*<0.0001*
G-carriers < AA	Sub-lobar	L	-23	8	13	596	4.77	*<0.0001*
Right frontoinsular seed (Salience Network)								
G-carriers < AA	Superior Frontal	R	18	54	22	191	3.85	*<0.0001*
Right dorsolateral prefrontal seed (Executive control network)								
G-carriers < AA	Superior Frontal	R	14	41	39	930	4.69	*<0.0001*
	Superior Frontal	R	17	44	27	s.c.	4.64	*<0.0001*
	Superior Temporal pole	R	36	12	-21	747	4.32	*<0.0001*
	Superior Temporal pole	R	38	12	-21	280	3.91	*<0.0001*

*SCN* structural covariance network, *DMN* default mode network, *s.c.* same cluster

Within the SCNs anchored to the left PCC, increased structural associations in the G carriers included the cerebellum and middle temporal clusters (Table 3; Fig. 2b, *blue lines*). Within the SCNs anchored to the left PCC seed, increased structural association in the A homozygotes was noted in the sublobar GM (Table 3; Fig. 2c, *green lines*).

For the right frontoinsular seed, an increased and significant correlation in the A homozygotes was found only in the superior frontal region (Table 3). For the ECN, increased correlations in the A homozygotes were found in the superior frontal and temporal pole (Table 3; Fig. 2d, *green lines*). For the aforementioned clusters, we found no dose-dependent effects in covariance strength when we compared the GG, GA, and AA genotype groups.

### Correlations of peak cluster volumes with neurobehavioral test scores

We then analyzed differences in the peak cluster volumes showing between-group interactions, of which the entorhinal seed anchored midfrontal peak clusters, frontoinsular seed-anchored superior-frontal peak cluster, and DLPFC-anchored superior-frontal peak cluster showed significantly greater volumes in the G carriers (Additional file 1: Table S13). For peak cluster volumes and neurobehavioral tests, we analyzed the clinical relationships of the G carriers (Table 4) and A homozygotes (Table 5) separately. In the G carriers, the significant peak clusters that correlated with the behavioral scores were mainly the PCC-anchored peak clusters. For the A homozygotes, significance was found in the DLPFC-anchored seed.

**Table 4:** Significant Relationships between peak cluster volumes and clinical parameters (Bcl-2 G-carriers)

Seed region	EC	PCC	PCC	PCC	dLPFC
Peak cluster	Superior frontal	cerebellum	cerebellum	Sub-lobar	Superior frontal
Covariance strength relationship	G < AA	G > AA	G > AA	G < AA	G < AA
MNI coordinate	(12,14,57)	(-33,-57,-21)	(-23,-55,-20)	(-23,8,14)	(14,41,39)
Mini-mental state examination	0.017	0.340	*0.261* ^a^	-0.171	*-0.227* ^a^
T1 to T4 trial scores	-0.108	*0.374* ^b^	*0.291* ^a^	-0.159	-0.112
30 second recalls	-0.049	*0.362* ^b^	0.193	-0.104	-0.112
10 minute recalls	-0.037	*0.259* ^a^	0.197	-0.087	-0.192
Modified Rey-Osterrieth Recall	-0.040	*0.273* ^a^	0.177	-0.056	-0.118
Modified Rey-Osterrieth Copy	-0.115	*0.270* ^a^	0.181	-0.020	-0.090
Visual object and Space Perception	-0.009	0.178	-0.007	-0.059	-0.084
Semantic fluency: Animal	-0.119	*0.256* ^a^	0.161	-0.083	-0.206
Boston Naming Test	0.093	*0.374* ^b^	0.214	-0.073	-0.144
Comprehension	-0.045	*0.317* ^b^	*0.321* ^b^	*-0.420* ^b^	-0.148
Digit backward	0.006	0.268	0.198	*-0.240* ^a^	-0.112
Stroop Interference Correct	*-0.255* ^a^	0.234	0.152	-0.085	-0.118
Design fluency	0.058	0.228	0.049	-0.015	-0.113
Trail making test time	0.066	*-0.230* ^a^	-0.076	0.201	0.194
Correct line in Trail making	-0.139	*0.286* ^a^	0.181	-0.163	-0.074
Neuropsychiatric Inventory	-0.068	-0.050	-0.064	-0.042	0.197

Numbers indicate Spearman correlation coefficient; ^a^*p* < 0.05; ^b^ *p* <0.01; *EC* entorinal cortex, *PCC* posterior cingulate cortex, *dLPFC* dorsolateral prefrontal cortex

**Table 5:** Significant Relationships between peak cluster volumes and clinical parameters (Bcl-2 AA)

Seed region	frontoinsular	dLPFC	dLPFC	dLPFC
Peak cluster	Superior frontal	Superior frontal	Superior temporal pole	temporal pole
Covariance strength relationship†	G < AA	G < AA	G < AA	G < AA
MNI coordinate	(18,54,22)	(14,41,39)	(36,12,-21)	(38,12,-21)
Mini-mental state examination	0.295	0.273	0.270	*0.411* ^a^
T1 to T4 trial scores	*0.441* ^a^	0.103	0.189	0.291
30 second recalls	0.328	0.335	*0.378* ^a^	*0.432* ^a^
10 minute recalls	0.342	0.259	0.147	0.207
Modified Rey-Osterrieth Recall	*0.454* ^a^	0.245	0.205	0.364
Modified Rey-Osterrieth Copy	*0.441* ^a^	0.176	0.184	0.169
Visual object and Space Perception	0.045	0.068	-0.113	0.121
Semantic fluency: Animal	0.328	0.144	0.098	-0.005
Boston Naming Test	0.120	0.152	0.159	0.146
Comprehension	0.261	0.098	-0.022	-0.029
Digit backward	0.202	0.175	0.373	0.061
Stroop Interference Correct	0.399	*0.537* ^b^	0.253	0.324
Design fluency	0.363	-0.045	0.219	0.403
Trail making test time	-0.186	-0.299	-0.324	-*0.520* ^b^
Correct line in Trail making	0.330	0.074	0.188	0.293
Neuropsychiatric Inventory	0.190	-0.131	-0.190	-0.038

Numbers indicate Spearman’s correlation coefficient; *MNI* Montreal Neurological Institute ^a^*p* < 0.05; ^b^ *p* <0.01; *dLPFC* dorsolateral prefrontal cortex

## Discussion

In this study, we analyzed differences between two Bcl-2 genotypes showing functional polymorphisms. The topography of the SCNs and clinical correlations also validated the different influences of the Bcl-2 genotype groups with regard to structural degeneration, which targeted the DMN midline subsystem in the G carriers and the ECN in the A homozygotes. The results support the hypothesis that network changes represent an endophenotype of the Bcl-2 polymorphism. In addition, the greater covariance strength of the AA genotype in all four networks suggests that covariance strength may serve as a putative biomarker for structural connectivity.

### Bcl-2 genotypes targeted different SCNs and modulated covariance strength

Current neuroscience research supports the hypothesis that brain networks in AD can be influenced by environmental and biological interactions not limited to amyloid toxicity [37] or network degenerative theory [38]. Accordingly, cognitive functions are highly reflective of neuronal network changes. A number of studies have highlighted how genetic variations may affect brain organization and connectivity patterns [39–42]. Our results provide insight into the Bcl-2 genetic variants and their possible interference with structural covariance.

Our SCN data suggest that genetic influences, in terms of network topography, were modulated differently by the two Bcl-2 genotypes. Spatial distributions revealed that the G carriers had more extended voxels in the DMN, whereas the A homozygotes had a larger structural association in the ECN. From a methodological perspective, the wider spatial extent in the G carriers may be partially driven by the larger number of cases. However, the correlation with clinical scores may validate the roles of DMN seed- or PCC-anchored peak clusters. The spatial extent of the SCN directly comparing the GG and GA genotype groups suggested that the SCN was not merely reflective of the number of cases. A further study with a larger sample size and an equal number of cases may help to elucidate our observations.

We previously reported that stronger covariance strength between seed and peak clusters indicates more intranetwork connections [43]. In a given genotype group, greater covariance strength may have indicated stronger associations between two interconnected clusters. In a degenerative model of AD, the pathological processes often lead to GM atrophy. Further longitudinal studies are needed to elucidate whether more intranetwork connections indicates faster degenerative processes.

For the subsystems of the DMN, our results suggest that the genetic weighting may be higher in the PCC-anchored midline DMN system in the G carriers, because it was the only SCN showing G → AA genotypes in covariance strength. Previous studies have indicated that the AA genotype may be a risk factor for a smaller intracranial volume or worse clinical phenotype [9, 21]. We also found that covariance strength was higher in the AA genotype group and that the functional associations may have been more localized in the ECN. Several peak cluster volumes were also significantly smaller in the AA genotype group (Additional file 1: Table S13) than in the G carriers.

The peak cluster represents regions that not only are anchored to the predefined seed but also show genotype group interactions in covariance strength. From a clinical point of view, the peak cluster may represent anatomical areas where genotype modulation occurs. Correlation analysis between the peak cluster volumes and clinical parameters was performed to establish the clinical significance; however, not all peak clusters were related to the clinical outcomes, and the peak clusters showing significant correlations were also different in each genotype group (Table 4 for G carriers; Table 5 for the AA genotype). With regard to the clinical implications, the peak cluster analysis suggested that the G carriers were more related to the SCN in the posterior brain regions, whereas the A homozygotes were more related to the anterior brain regions.

### Clinical significance of executive control network in Bcl-2 A homozygotes

In this study, seed regions, covariance strength, and peak clusters all supported the unique genetic effect of A homozygotes in the ECN. The ECN in our qualitative data consisted of the classic DLPFC and the parietal cortex [13], and the increased A > G interactions in the cortical hubs with associated clinical correlations supported its functional role. The Bcl-2 AA genotype has been associated with a reduced level of Bcl-2 [6], whereas lower Bcl-2 protein and mRNA levels have been reported in the frontal cortex in bipolar disorders [7]. Our observations may echo these findings [21] and support the genetic associations of the AA genotype via its modulation of GM in the ECN in patients with AD. In this study, we also tested whether the A allele may have a dose-dependent effect on the covariance strength. Although the results were not significant, this insignificance may not fully reject the possible functional effect of the A allele, because only a small number of cases were included in this study, even though they fulfilled the Hardy-Weinberg principle.

### Increased covariance strength in the DMN midtemporal subsystem in A homozygotes

Although the medial frontal cortex has been shown to play a role in learning associations and the DLPFC has been shown to play a role in executive function, the coactivation of these prefrontal neural resources in AD has also been shown to compensate for posterior degenerative processes [44]. Functional disconnection between the entorhinal seed and midfrontal regions, however, has been observed in AD [45]. Although strong connections between the prefrontal cortex and the hippocampus may reinforce the learning and memory consolidation process, the integrated activity between these two regions tends to break down early in AD. If entorhinal-prefrontal connections represent compensatory mechanisms in the early stage of AD, the differences in covariance strength between two Bcl-2 genotype groups may have resulted in different brain reserves.

### Bcl-2 G carriers and the relationships with the two DMN subsystems

The genetic influence in the G carriers may have resulted in different genetic associations in the two DMN subsystems. The G genotype may have modulated the midline DMN subsystem because the PCC-anchored peak cluster volumes were related to more cognitive test domains showing statistical significance. The PCC-anchored DMN was also the only network to show greater covariance strength in the G carriers. Taken together, these findings suggest the unique role of the Bcl-2 G genotype group in the PCC-anchored DMN.

In AD, the salient cognitive dysfunction is episodic memory impairment, which has been reported to be strongly associated with the hippocampal/entorhinal volume and the midtemporal DMN subsystem [46, 47]. The significant correlations of the entorhinal seed volumes and the clinical scores highlight the primary role of the entorhinal seed in the G carriers (Table 2). In contrast, the entorhinal-anchored peak clusters were not predictive of cognitive scores.

### Salience network and Bcl-2 G carriers

In bipolar disorder [21], a link between the anterior cingulate cortex, one of the key hubs in the salience network, and the salience network and Bcl-2 protein expression may exist. The role of the salience network has been suggested to support the processing of diverse homeostatically relevant internal and external stimuli [48]. For the salience network analysis in this study, the seed frontoinsular region predicted scores on the Stroop test and the Trail Making Test, which were predominantly classified as external stimuli. Although the salience network was originally discovered to be related to internal emotional states [13], our study did not establish a clinical role of the salience network in mediating NPI scores in either the G carriers or A homozygotes.

### Study limitations

Direct comparisons between the G carriers and A homozygotes showed between-group differences in NPI total scores, aggression, and sleep subscores. However, our network analysis did not find any relationships linking the SCNs to any of the significant clinical results. Because our study design used a seed-based approach, it is possible that the changes were mediated by different functional networks. The use of independent component analysis [49] may help to overcome this limitation. Another important limitation of this study is that we did not include a control group, and the inclusion of a control group may have helped to elucidate whether the Bcl-2 functional polymorphisms exerted similar GM modulation patterns in healthy elderly subjects. However, associations between rs956572 functional polymorphisms and regional GM volume and functional states in healthy subjects have been reported [8, 9, 21, 50]. Given the differences in methods, sample sizes, and populations analyzed, it is difficult to compare our results directly with these reports. However, we found regional similarities in this study. Another potential limitation is that our seed-based analysis emphasized the SCNs that showed positive correlations with the seeds. Because the aim of this study was to test the hypothesis of the risk of different genotype groups, anticorrelation patterns suggestive of a compensatory process in each genotype group were not explored. Because clinical significance was established in restricted nodes showing covariance interactions, whether the seed or peak clusters imparted an equal amount of information within the whole network remains an important issue that needs to be investigated in future studies. Last, structural covariance data cannot be directly referred to as a connectivity or degenerative biomarker, although the patterns of SCN have been shown to mirror those of intrinsic connectivity patterns in healthy control subjects [51]. Further studies with longitudinal cohorts are needed to validate the interpretation regarding greater covariance strength in the AA genotype and faster degeneration. In addition, to elucidate the functional effect of the A allele observed in this study, a larger sample cohort is required in future studies so that direct comparisons of the AA and GG genotypes with equal and adequate case numbers are possible.

## Conclusions

In summary, the SCN analysis and covariance strength interactions support the genetic influences of the Bcl-2 rs956572 functional polymorphism on the SCN in the early stage of AD. We show a greater genetic influence in the A homozygotes on the ECN, whereas the genetic modulation in the G carriers was seen in the PCC-anchored midline DMN subsystem. We will investigate the biological meaning of covariance strength in future studies with longitudinal follow-up.

## Additional files

Additional file 1: Supplementary **Tables S1–S13**. (DOCX 67 kb)

Additional file 2: Figure S1. Structural covariance networks from four seed regions in GG, GA, and A homozygotes. According to the case numbers, the actual T value in AA was 5.529, GA = 4.924, and GG = 6.4612. (BMP 7288 kb)

## Abbreviations

AD: Alzheimer’s disease
ApoE: Apolipoprotein E
BCL: B-cell chronic lymphocytic leukemia/lymphoma-2
DLPFC: Dorsolateral prefrontal cortex
DMN: Default mode network
ECN: Executive control network
FDR: False discovery rate
GM: Gray matter
MMSE: Mini Mental State Examination
MNI: Montreal Neurological Institute
MRI: Magnetic resonance imaging
mRNA: Messenger RNA
NPI: Neuropsychiatric Inventory
PCC: Posterior cingulate cortex
ROI: Region of interest
SCN: Structural covariance network
