# Supplementary material for: Associations of Bcl-2 rs956572 genotype groups in the structural covariance network in early-stage Alzheimer’s disease
Source: Alzheimers Res Ther. 2018 Feb 8;10:17. doi: 10.1186/s13195-018-0344-4 (PMC5806294; doi:10.1186/s13195-018-0344-4)
Supplement: Supplementary file 1 — Supplementary Tables S1–S13. (DOCX 67 kb) [file 13195_2018_344_MOESM1_ESM.docx]

**Additional file 1**

**Table S1.** Structural covariance network for all patients with right entorhinal cortex as seed.

| **Main Cluster** | **Peak regions** |  | **Stereotaxic coordinates** | | | **Extent** | **Max T** | **P-value** |
| --- | --- | --- | --- | --- | --- | --- | --- | --- |
|  |  | Side | x | y | z |  |  |  |
| **ParaHippocampal** |  | **R** | **27** | **-11** | **-29** | **95539** | **25.03** | **<0.0001** |
|  | ParaHippocampal | L | -23 | -11 | -27 | s.c. | 8.6 | <0.0001 |
| **Parietal_Sup** |  | **L** | **-18** | **-75** | **54** | **3302** | **4.08** | **<0.0001** |
|  | Parietal_Sup | L | -20 | -53 | 69 | s.c. | 4.07 | <0.0001 |
|  | Parietal_Sup | L | -29 | -54 | 56 | s.c. | 3.85 | <0.0001 |
| **Postcentral** |  | **R** | **27** | **-32** | **53** | **6065** | **4.07** | **<0.0001** |
|  | Frontal_Mid | R | 35 | 23 | 41 | s.c. | 3.9 | <0.0001 |
|  | Cingulum_Mid | R | 11 | -38 | 45 | s.c. | 3.84 | <0.0001 |
| **Frontal_Sup** |  | **L** | **-27** | **48** | **0** | **1072** | **4.06** | **<0.0001** |
|  | Frontal_Mid | L | -33 | 47 | 15 | s.c. | 3.2 | 0.001 |
|  | Frontal_Mid_Orb | L | -21 | 54 | -9 | s.c. | 2.65 | 0.005 |
| **Cingulum_Mid** |  | **L** | **-9** | **-32** | **45** | **1685** | **3.66** | **<0.0001** |
|  | Paracentral_Lobule | L | -14 | -29 | 63 | s.c. | 2.91 | 0.002 |
| **Frontal_Sup_Medial** |  | **L** | **-3** | **63** | **8** | **353** | **3.35** | **0.001** |
| **Medial Frontal Gyrus** |  | **L** | **-12** | **-12** | **60** | **377** | **3.19** | **0.001** |
|  | Supp_Motor_Area | L | -8 | -5 | 74 | s.c. | 2.81 | 0.003 |
| **Angular** |  | **L** | **-41** | **-62** | **27** | **262** | **3.11** | **0.001** |
| **Cerebelum_9** |  | **L** | **-14** | **-54** | **-42** | **116** | **2.88** | **0.002** |
| **Cerebelum_8** |  | **L** | **-26** | **-53** | **-47** | **s.c.** | **2.54** | **0.006** |
|  | Cerebelum_8 | L | -24 | -62 | -45 | s.c. | 2.5 | 0.007 |
| **Superior Frontal Gyrus** |  | **R** | **23** | **62** | **-20** | **141** | **2.67** | **0.004** |
| **Frontal_Mid** |  | **L** | **-29** | **27** | **35** | **132** | **2.56** | **0.006** |
|  | Frontal_Mid | L | -21 | 44 | 30 | s.c. | 2.45 | 0.008 |
|  | Frontal_Mid | L | -24 | 35 | 30 | s.c. | 2.35 | 0.01 |

Peak regions are within the Main cluster

Max T is the maximum T statistic for each local maximum. P<0.05 based on non-stationary cluster-extent False discovery rate correction.

**Table S2.** Structural covariance network for all patients with left posterior cingulate as seed.

| **Main Cluster** | **Peak regions** |  | **Stereotaxic coordinates** | | | **Extent** | **Max T** | **P-value** |
| --- | --- | --- | --- | --- | --- | --- | --- | --- |
|  |  | Side | x | y | z |  |  |  |
| Cingulum_Mid |  | L | -2 | -36 | 35 | 212363 | 40.53 | **<0.0001** |
|  | Cingulum_Mid | L | 6 | 11 | 41 | s.c. | 9.58 | <0.0001 |
|  | Angular | L | -48 | -69 | 33 | s.c. | 9.22 | <0.0001 |

Peak regions are within the Main cluster

Max T is the maximum T statistic for each local maximum. P<0.05 based on non-stationary cluster-extent False discovery rate correction.

**Table S3.** Structural covariance network for all patients with right frontoinsularcortex as seed.

| **Main Cluster** | **Peak regions** |  | **Stereotaxic coordinates** | | | **Extent** | **Max T** | **P-value** |
| --- | --- | --- | --- | --- | --- | --- | --- | --- |
|  |  | Side | x | y | z |  |  |  |
| **Frontal_Inf_Orb** |  | **R** | **38** | **27** | **-11** | **96366** | **23.69** | **<0.0001** |
|  | Insula | L | -29 | 20 | -6 | s.c. | 6.74 | <0.0001 |
|  | Frontal_Inf_Orb | R | 27 | 32 | -18 | s.c. | 6.5 | <0.0001 |
| **Precuneus** |  | **R** | **11** | **-65** | **30** | **2108** | **4.07** | **<0.0001** |
|  | Precuneus | R | 11 | -59 | 20 | s.c. | 3.68 | <0.0001 |
|  | Lingual | R | 21 | -54 | -5 | s.c. | 2.58 | 0.006 |
| **Frontal_Sup** |  | **R** | **21** | **-2** | **71** | **596** | **3.29** | **0.001** |
|  | Frontal_Sup | R | 23 | -9 | 63 | s.c. | 3.29 | 0.001 |
| **Cingulum_Ant** |  | **L** | **-8** | **27** | **30** | **333** | **2.85** | **0.003** |
|  | Frontal_Sup_Medial | L | -8 | 21 | 39 | s.c. | 2.78 | 0.003 |
| **Cerebelum_6** |  | **L** | **-14** | **-68** | **-24** | **105** | **2.8** | **0.003** |
| **Cerebelum_6** |  | **R** | **18** | **-62** | **-21** | **205** | **2.72** | **0.004** |
|  | Fusiform | R | 32 | -63 | -15 | s.c. | 2.61 | 0.005 |
| **Cingulate Gyrus** |  |  | **6** | **-36** | **26** | **103** | **2.71** | **0.004** |

Peak regions are within the Main cluster

Max T is the maximum T statistic for each local maximum. P<0.05 based on non-stationary cluster-extent False discovery rate correction.

**Table S4.** Structural covariance network for all patients with right dorsolateral prefrontal as seed.

| **Main Cluster** | **Peak regions** |  | **Stereotaxic coordinates** | | | **Extent** | **Max T** | **P-value** |
| --- | --- | --- | --- | --- | --- | --- | --- | --- |
|  |  | Side | x | y | z |  |  |  |
| **Frontal_Mid** |  | **R** | **42** | **36** | **20** | **92091** | **20.08** | **<0.0001** |
|  | Frontal_Mid | R | 42 | 44 | 9 | s.c. | 7.14 | <0.0001 |
|  | Frontal_Sup_Medial | R | 12 | 50 | 6 | s.c. | 6.67 | <0.0001 |
| **Postcentral** |  | **L** | **-47** | **-30** | **47** | **723** | **3.95** | **<0.0001** |
|  | Parietal_Inf | L | -45 | -38 | 41 | s.c. | 2.77 | 0.003 |
| **Occipital_Mid** |  | **L** | **-32** | **-80** | **14** | **768** | **3.78** | **<0.0001** |
|  | Occipital_Mid | L | -26 | -86 | 17 | s.c. | 3.27 | 0.001 |
|  | Occipital_Mid | L | -42 | -75 | 9 | s.c. | 2.58 | 0.006 |
| **Precuneus** |  | **R** | **12** | **-68** | **33** | **320** | **3.28** | **0.001** |
| **Frontal_Sup** |  | **R** | **21** | **-12** | **65** | **221** | **3.27** | **0.001** |
| **Parahippocampa Gyrus** |  | **L** | **-12** | **-33** | **0** | **826** | **3.22** | **0.001** |
|  | Thalamus | R | 2 | -21 | 3 | s.c. | 3.15 | 0.001 |

Peak regions are within the Main cluster

Max T is the maximum T statistic for each local maximum. P<0.05 based on non-stationary cluster-extent False discovery rate correction.

**Table S5.** Structural covariance network for G-carriers with right entorhinal cortex as seed.

| **Main Cluster** | **Peak regions** |  | **Stereotaxic coordinates** | | | **Extent** | **Max T** | **P-value** |
| --- | --- | --- | --- | --- | --- | --- | --- | --- |
|  |  | Side | x | y | z |  |  |  |
| **ParaHippocampal** |  | **R** | **27** | **-11** | **-29** | **31223** | **22.23** | **<0.0001** |
|  | Temporal_Inf | R | 57 | 0 | -33 |  | 7.09 | <0.0001 |
| **ParaHippocampal** |  | **L** | **-23** | **-9** | **-27** | **24817** | **9.06** | **<0.0001** |
|  | ParaHippocampal | L | -21 | 3 | -26 |  | 8.32 | <0.0001 |
|  | Temporal_Pole_Mid | L | -30 | 5 | -39 |  | 5.69 | <0.0001 |
| **Occipital_Mid** |  | **R** | **38** | **-75** | **20** | **2067** | **4.64** | **<0.0001** |
|  | Occipital_Mid | R | 39 | -69 | 33 |  | 4.52 | <0.0001 |
|  | Occipital_Mid | R | 39 | -78 | 0 |  | 3.73 | <0.0001 |
| **Frontal_Mid_Orb** |  | **L** | **-27** | **48** | **-2** | **622** | **4.22** | **<0.0001** |
|  | Frontal_Mid_Orb | L | -23 | 56 | -6 |  | 2.83 | 0.003 |
| **Occipital_Sup** |  | **L** | **-14** | **-93** | **11** | **135** | **3.8** | **<0.0001** |
| **Postcentral** |  | **L** | **-51** | **-23** | **27** | **151** | **3.78** | **<0.0001** |
| **SupraMarginal** |  | **R** | **54** | **-38** | **41** | **413** | **3.62** | **<0.0001** |
| **Parietal_Sup** |  | **L** | **-30** | **-51** | **56** | **488** | **3.41** | **0.001** |
|  | Parietal_Sup | L | -20 | -53 | 69 |  | 3.22 | 0.001 |
|  | Parietal_Sup | L | -18 | -62 | 63 |  | 3.17 | 0.001 |
| **Frontal_Sup** |  | **R** | **29** | **29** | **50** | **526** | **3.39** | **0.001** |
|  | Frontal_Mid | R | 33 | 23 | 42 |  | 3.09 | 0.001 |
|  | Frontal_Mid | R | 38 | 30 | 38 |  | 2.82 | 0.003 |
| **Inferior Occipital Gyrus** |  | **L** | **-36** | **-92** | **-9** | **693** | **3.37** | **0.001** |
| **Cingulum_Mid** |  | **R** | **9** | **-30** | **36** | **176** | **3.34** | **0.001** |
| **Frontal_Sup_Orb** |  | **R** | **35** | **62** | **-6** | **202** | **3.3** | **0.001** |
| **Rolandic_Oper** |  | **L** | **-47** | **-12** | **17** | **344** | **3.1** | **0.001** |
| **Precentral** |  | **R** | **30** | **-21** | **60** | **217** | **3.02** | **0.002** |
|  | Postcentral | R | 27 | -30 | 53 |  | 3 | 0.002 |

Peak regions are within the Main cluster

Max T is the maximum T statistic for each local maximum. P<0.05 based on non-stationary cluster-extent False discovery rate correction.

**Table S6.** Structural covariance network for A-homozygotes with right entorhinal cortex as seed.

| **Main Cluster** | **Peak regions** |  | Stereotaxic coordinates | | | Extent | Max T | P-value (FDR-corr). |
| --- | --- | --- | --- | --- | --- | --- | --- | --- |
|  |  | Side | x | y | z |  |  |  |
| **ParaHippocampal** |  | **R** | **29** | **-11** | **-29** | **2678** | **12.1** | **<0.0001** |
|  | Fusiform | R | 39 | -21 | -20 | s.c. | 4.91 | <0.0001 |
|  | Fusiform | R | 39 | -39 | -17 | s.c. | 4.63 | <0.0001 |
| **Temporal_Inf** |  | **L** | **-39** | **-36** | **-17** | **366** | **5.93** | **<0.0001** |
|  | Fusiform | L | -35 | -48 | -15 | s.c. | 3.69 | 0.001 |
| **Occipital_Mid** |  | **L** | **-41** | **-66** | **24** | **770** | **5.76** | **<0.0001** |
|  | Occipital_Mid | L | -39 | -78 | 36 | s.c. | 4.08 | <0.0001 |
|  | Occipital_Mid | L | -30 | -75 | 41 | s.c. | 4 | <0.0001 |
| **Occipital_Inf** |  | **L** | **-38** | **-75** | **-12** | **108** | **5.62** | **<0.0001** |
| **Parietal Lobe** |  | **R** | **21** | **-41** | **54** | **484** | **4.96** | **<0.0001** |
|  | Postcentral | R | 23 | -36 | 63 | s.c. | 3.68 | 0.001 |
| **ParaHippocampal** |  | **L** | **-15** | **-35** | **-6** | **310** | **4.76** | **<0.0001** |
|  | Hippocampus | L | -14 | -41 | 3 | s.c. | 4.49 | <0.0001 |
|  | Hippocampus | L | -20 | -29 | -12 | s.c. | 4.35 | <0.0001 |
| **Precuneus** |  | **L** | **-8** | **-78** | **51** | **728** | **4.64** | **<0.0001** |
|  | Cuneus | L | -3 | -71 | 26 | s.c. | 4.36 | <0.0001 |
|  | Precuneus | L | -6 | -69 | 60 | s.c. | 4.24 | <0.0001 |
| **Frontal_Sup** |  | **L** | **-14** | **35** | **38** | **298** | **4.55** | **<0.0001** |
|  | Frontal_Sup | L | -18 | 45 | 32 | s.c. | 4.19 | <0.0001 |
|  | Frontal_Mid | L | -23 | 35 | 41 | s.c. | 4 | <0.0001 |
| **Frontal_Mid** |  | **L** | **-30** | **39** | **20** | **503** | **4.55** | **<0.0001** |
|  | Frontal_Mid | L | -29 | 30 | 32 | s.c. | 4.06 | <0.0001 |
| **Postcentral** |  | **L** | **-59** | **-12** | **23** | **189** | **4.27** | **<0.0001** |
| **Temporal_Inf** |  | **L** | **-44** | **-8** | **-27** | **236** | **4.16** | **<0.0001** |
|  | Temporal_Inf | L | -47 | -15 | -26 | s.c. | 4.06 | <0.0001 |
|  | Temporal_Inf | L | -54 | -35 | -26 | s.c. | 3.9 | <0.0001 |
| **Rolandic_Oper** |  | **L** | **-44** | **0** | **11** | **131** | **4.08** | **<0.0001** |
|  | Rolandic_Oper | L | -39 | -9 | 12 | s.c. | 3.96 | <0.0001 |
| **Temporal_Mid** |  | **L** | **-59** | **-36** | **0** | **196** | **4.04** | **<0.0001** |

Peak regions are within the Main cluster

Max T is the maximum T statistic for each local maximum. P<0.05 based on non-stationary cluster-extent False discovery rate correction.

**Table S7.** Structural covariance network for G-carriers with left posterior cingulate as seed.

| **Main Cluster** | **Peak regions** |  | **Stereotaxic coordinates** | | | **Extent** | **Max T** | **P-value** |
| --- | --- | --- | --- | --- | --- | --- | --- | --- |
|  |  | Side | x | y | z |  |  |  |
| **Cingulum_Mid** |  | **L** | **-2** | **-36** | **35** | **203166** | **31.63** | **<0.0001** |
|  | Angular | L | -47 | -71 | 36 | s.c. | 9.07 | <0.0001 |
| **Fusiform** |  | **L** | **-20** | **-2** | **-44** | **193** | **2.7** | **0.004** |

Peak regions are within the Main cluster

Max T is the maximum T statistic for each local maximum. P<0.05 based on non-stationary cluster-extent False discovery rate correction.

**Table S8.** Structural covariance network for A-homozygotes with left posterior cingulate as seed.

| **Main Cluster** | **Peak regions** |  | **Stereotaxic coordinates** | | | **Extent** | **Max T** | **P-value** |
| --- | --- | --- | --- | --- | --- | --- | --- | --- |
|  |  | Side | x | y | z |  |  |  |
| **Cingulum_Mid** |  | **L** | **-3** | **-36** | **35** | **24741** | **26.9** | **<0.0001** |
|  | Precuneus | L | 0 | -53 | 48 | s.c. | 6.63 | <0.0001 |
|  | Cingulum_Mid | R | 5 | 17 | 39 | s.c. | 5.76 | <0.0001 |
| **Frontal_Inf_Oper** |  | **L** | **-47** | **11** | **27** | **2905** | **5.16** | **<0.0001** |
|  | Frontal_Mid | L | -41 | 29 | 30 | s.c. | 4.58 | <0.0001 |
|  | Rolandic_Oper | L | -57 | -6 | 12 | s.c. | 4.47 | <0.0001 |
| **Precentral** |  | **R** | **48** | **9** | **30** | **1320** | **4.09** | **<0.0001** |
|  | Frontal_Mid | R | 29 | 35 | 45 | s.c. | 4 | <0.0001 |
|  | Frontal_Sup | R | 26 | 27 | 48 | s.c. | 3.91 | <0.0001 |
| **Frontal_Sup_Orb** |  | **R** | **14** | **68** | **-2** | **393** | **3.74** | **<0.0001** |
|  | Frontal_Sup_Orb | R | 29 | 63 | -3 | s.c. | 3.19 | 0.002 |
| **Angular** |  | **L** | **-50** | **-63** | **36** | **290** | **3.73** | **<0.0001** |
| **Frontal_Mid** |  | **R** | **27** | **48** | **32** | **188** | **3.62** | **0.001** |
|  | Frontal_Sup | R | 18 | 50 | 32 | s.c. | 3.2 | 0.002 |
| **Sub-lobar** |  | **L** | **-8** | **0** | **-2** | **154** | **3.6** | **0.001** |
|  | Thalamus | L | -3 | -9 | 0 | s.c. | 3.03 | 0.003 |
| **SupraMarginal** |  | **R** | **54** | **-35** | **30** | **149** | **3.52** | **0.001** |
| **Temporal_Inf** |  | **L** | **-50** | **0** | **-42** | **136** | **3.42** | **0.001** |
| **Rolandic_Oper** |  | **R** | **60** | **-2** | **8** | **220** | **3.37** | **0.001** |
|  | Rolandic_Oper | R | 57 | 3 | 17 | s.c. | 2.97 | 0.003 |
| **Caudate** |  | **L** | **-11** | **17** | **8** | **216** | **3.33** | **0.001** |
| **Caudate** |  | **R** | **11** | **0** | **17** | **232** | **3.32** | **0.001** |

Peak regions are within the Main cluster

Max T is the maximum T statistic for each local maximum. P<0.05 based on non-stationary cluster-extent False discovery rate correction.

**Table S9.** Structural covariance network for G-carriers with right frontoinsularcortex as seed.

| **Main Cluster** | **Peak regions** |  | **Stereotaxic coordinates** | | | **Extent** | **Max T** | **P-value** |
| --- | --- | --- | --- | --- | --- | --- | --- | --- |
|  |  | Side | x | y | z |  |  |  |
| **Frontal_Inf_Orb** |  | **R** | **38** | **27** | **-11** | **12769** | **18.54** | **<0.0001** |
|  | Frontal_Inf_Orb | R | 29 | 32 | -20 | s.c. | 5.2 | <0.0001 |
| **Insula** |  | **L** | **-29** | **20** | **-5** | **2077** | **5.79** | **<0.0001** |
|  | Cingulum_Ant | L | -9 | 41 | -6 | s.c. | 3.66 | <0.0001 |
| **Temporal_Inf** |  | **L** | **-39** | **-15** | **-36** | **1870** | **4.62** | **<0.0001** |
|  | Temporal_Pole_Mid | L | -18 | 6 | -41 | s.c. | 4.45 | <0.0001 |
|  | Fusiform | L | -29 | -26 | -29 | s.c. | 3.82 | <0.0001 |
| **Temporal_Mid** |  | **L** | **-54** | **-18** | **-18** | **1460** | **4.52** | **<0.0001** |
|  | Temporal_Mid | L | -51 | -5 | -23 | s.c. | 3.87 | <0.0001 |
|  | Temporal_Mid | L | -45 | 5 | -24 | s.c. | 3.65 | <0.0001 |
| **Cerebelum_7b** |  | **L** | **-17** | **-72** | **-38** | **220** | **4.07** | **<0.0001** |
| **Cerebelum_6** |  | **L** | **-12** | **-69** | **-24** | **119** | **3.75** | **<0.0001** |
| **SupraMarginal** |  | **R** | **63** | **-38** | **41** | **163** | **3.74** | **<0.0001** |
| **Angular** |  | **R** | **56** | **-56** | **26** | **443** | **3.59** | **<0.0001** |
|  | Temporal_Sup | R | 57 | -41 | 21 | s.c. | 3.39 | 0.001 |
| **Occipital_Mid** |  | **L** | **-41** | **-75** | **11** | **226** | **3.58** | **<0.0001** |
| **Temporal_Mid** |  | **L** | **-45** | **-62** | **2** | **141** | **3.55** | **<0.0001** |
|  | Temporal_Mid | L | -51 | -53 | 2 | s.c. | 3.38 | 0.001 |
|  | Temporal_Mid | L | -54 | -42 | 9 | 233 | 3.46 | 0.001 |
| **Temporal_Mid** |  | **L** | **-45** | **-50** | **18** | **s.c.** | **3.24** | **0.001** |
| **Temporal_Mid** |  | **R** | **50** | **-65** | **12** | **182** | **3.34** | **0.001** |
|  | Temporal_Mid | R | 51 | -54 | 12 | s.c. | 3.09 | 0.001 |
|  | Temporal_Mid | R | 44 | -71 | 9 | s.c. | 2.97 | 0.002 |

Peak regions are within the Main cluster

Max T is the maximum T statistic for each local maximum. P<0.05 based on non-stationary cluster-extent False discovery rate correction.

**Table S10.** Structural covariance network for A-homozygotes with right frontoinsularcortex as seed.

| **Main Cluster** | **Peak regions** |  | **Stereotaxic coordinates** | | | **Extent** | **Max T** | **P-value** |
| --- | --- | --- | --- | --- | --- | --- | --- | --- |
|  |  | Side | x | y | z |  |  |  |
| **Frontal_Inf_Orb** |  | **R** | **38** | **26** | **-11** | **11411** | **16.1** | **<0.0001** |
|  | Frontal_Sup | R | 15 | 57 | 15 | s.c. | 5.39 | <0.0001 |
|  | Frontal_Inf_Tri | R | 47 | 33 | 6 | s.c. | 5.26 | <0.0001 |
| **Frontal_Sup** |  | **L** | **-12** | **50** | **21** | **1250** | **7.6** | **<0.0001** |
|  | Frontal_Sup | L | -20 | 56 | 29 | s.c. | 4.6 | <0.0001 |
|  | Frontal_Sup_Medial | L | -14 | 53 | 12 | s.c. | 4.37 | <0.0001 |
| **Frontal_Inf_Orb** |  | **L** | **-36** | **18** | **-15** | **4196** | **6.27** | **<0.0001** |
|  | Frontal_Inf_Tri | L | -47 | 23 | 12 | s.c. | 6.06 | <0.0001 |
|  | Frontal_Inf_Tri | L | -39 | 24 | 5 | s.c. | 5.45 | <0.0001 |
| **Temporal_Mid** |  | **L** | **-48** | **-26** | **0** | **716** | **6.1** | **<0.0001** |
|  | Temporal_Sup | L | -47 | -41 | 11 | s.c. | 3.49 | 0.001 |
| **Frontal_Mid_Orb** |  | **L** | **-33** | **60** | **-6** | **143** | **4.01** | **<0.0001** |
|  | Frontal_Mid_Orb | L | -36 | 48 | -9 | s.c. | 3.58 | 0.001 |
| **Frontal_Inf_Oper** |  | **R** | **54** | **9** | **21** | **111** | **3.78** | **<0.0001** |
| **Heschl** |  | **R** | **39** | **-23** | **11** | **263** | **3.71** | **0.001** |
| **Cuneus** |  | **R** | **12** | **-69** | **32** | **231** | **3.67** | **0.001** |
| **Temporal_Inf** |  | **L** | **-50** | **-36** | **-20** | **135** | **3.59** | **0.001** |
|  | Temporal_Inf | L | -47 | -36 | -27 | s.c. | 3.37 | 0.001 |
| **Hippocampus** |  | **R** | **29** | **-15** | **-14** | **100** | **3.47** | **0.001** |
|  | Hippocampus | R | 21 | -11 | -20 | s.c. | 3.19 | 0.002 |

Peak regions are within the Main cluster

Max T is the maximum T statistic for each local maximum. P<0.05 based on non-stationary cluster-extent False discovery rate correction.

**Table S11.** Structural covariance network for G-carriers with right dorsolateral prefrontal as seed.

| **Main Cluster** | **Peak regions** |  | **Stereotaxic coordinates** | | | **Extent** | **Max T** | **P-value** |
| --- | --- | --- | --- | --- | --- | --- | --- | --- |
|  |  | Side | x | y | z |  |  |  |
| **Frontal_Mid** |  | **R** | **42** | **36** | **20** | **1975** | **18.21** | **<0.0001** |
|  | Frontal_Mid | R | 42 | 44 | 6 | s.c. | 5.21 | <0.0001 |
|  | Frontal_Mid | R | 44 | 14 | 47 | s.c. | 3.73 | <0.0001 |
| **Cingulum_Ant** |  | **R** | **11** | **50** | **8** | **396** | **5.57** | **<0.0001** |
| **Frontal_Sup_Orb** |  | **R** | **24** | **32** | **-15** | **368** | **5.08** | **<0.0001** |
| **Cingulum_Mid** |  | **R** | **8** | **5** | **38** | **425** | **4.4** | **<0.0001** |
|  | Cingulum_Mid | R | 8 | 20 | 32 | s.c. | 4.21 | <0.0001 |
|  | Cingulum_Ant | R | 8 | 32 | 24 | s.c. | 3.81 | <0.0001 |
| **Frontal_Mid_Orb** |  | **L** | **-24** | **30** | **-18** | **300** | **4.3** | **<0.0001** |
| **Frontal_Inf_Orb** |  | **L** | **-39** | **21** | **-12** | **208** | **4.2** | **<0.0001** |
| **Temporal_Sup** |  | **R** | **48** | **-30** | **15** | **100** | **3.71** | **<0.0001** |
|  | Temporal_Sup | R | 41 | -33 | 11 | s.c. | 3.48 | <0.0001 |

Peak regions are within the Main cluster

Max T is the maximum T statistic for each local maximum. P<0.05 based on non-stationary cluster-extent False discovery rate correction.

**Table S12.** Structural covariance network for A-homozygotes with right dorsolateral prefrontal as seed.

| **Main Cluster** | **Peak regions** |  | **Stereotaxic coordinates** | | | **Extent** | **Max T** | **P-value** |
| --- | --- | --- | --- | --- | --- | --- | --- | --- |
|  |  | Side | x | y | z |  |  |  |
| **Frontal_Mid** |  | **R** | **42** | **35** | **20** | **76381** | **10.13** | **<0.0001** |
|  | Insula | L | -38 | 18 | -8 | s.c. | 7.08 | <0.0001 |
|  | Brodmann area 9 | **R** | **4.5** | **54** | **19** | **s.c** | **4.39** | <0.0001 |
| **Occipital_Mid** |  | **L** | **-38** | **-81** | **15** | **1201** | **3.95** | **<0.0001** |
|  | Occipital_Mid | L | -30 | -81 | 11 | s.c. | 3.82 | <0.0001 |
|  | Occipital_Mid | L | -39 | -75 | 21 | s.c. | 3.57 | 0.001 |
| **Precentral** |  | **R** | **42** | **-8** | **47** | **175** | **3.9** | **<0.0001** |
|  | Precentral | R | 39 | 0 | 47 | s.c. | 2.76 | 0.005 |
| **Precuneus** |  | **L** | **-9** | **-56** | **24** | **927** | **3.75** | **<0.0001** |
| **Postcentral** |  | **L** | **-47** | **-30** | **48** | **216** | **3.6** | **0.001** |
|  | Postcentral | L | -36 | -33 | 42 | s.c. | 2.72 | 0.006 |
| **Hippocampus** |  | **L** | **-26** | **-30** | **-9** | **135** | **3.52** | **0.001** |
| **Occipital_Inf** |  | **L** | **-24** | **-86** | **-6** | **214** | **3.35** | **0.001** |
| **SupraMarginal** |  | **R** | **44** | **-39** | **41** | **136** | **3.25** | **0.002** |
|  | Parietal_Inf | R | 44 | -47 | 48 | s.c. | 2.97 | 0.003 |
| **Temporal_Sup** |  | **R** | **60** | **-50** | **18** | **391** | **3.11** | **0.002** |
|  | Temporal_Sup | R | 59 | -41 | 15 | s.c. | 3 | 0.003 |
|  | Temporal_Mid | R | 60 | -39 | 5 | s.c. | 2.58 | 0.008 |
| **Parietal_Inf** |  | **L** | **-44** | **-47** | **50** | **264** | **3.06** | **0.003** |
|  | Parietal_Inf | L | -53 | -51 | 38 | s.c. | 2.9 | 0.004 |
| **Precuneus** |  | **R** | **9** | **-69** | **35** | **254** | **2.99** | **0.003** |
|  | Precuneus | R | 11 | -71 | 45 | s.c. | 2.76 | 0.005 |

Peak regions are within the Main cluster

Max T is the maximum T statistic for each local maximum. P<0.05 based on non-stationary cluster-extent False discovery rate correction.

**Table S13.** Comparisons between peak cluster volumes in two Bcl-2 genotype groups

| Bcl-2 genotype groups | G-carriers | | AA | |
| --- | --- | --- | --- | --- |
|  | Mean | S.D. | Mean | S.D. |
| Seed : Right entorhinal cortex |  |  |  |  |
| Mid-Occipital (39,-66,24) | 0.56 | 0.17 | 0.55 | 0.18 |
| Mid-frontal (29,29,34) | 0.53* | 0.11 | 0.50 | 0.21 |
| Superior Frontal Gyrus (12,14,57) | 0.42 | 0.15 | 0.45 | 0.20 |
| Seed : left PCC |  |  |  |  |
| Left Cerebellum (-33,-57,-21) | 0.66 | 0.14 | 0.69 | 0.15 |
| Left Cerebellum (-23,-55,-20) | 0.79 | 0.12 | 0.83 | 0.13 |
| Sub-lobar (-23,8,13) | 0.25 | 0.10 | 0.23 | 0.10 |
| Seed : Right Fronto-insular |  |  |  |  |
| Superior Frontal (18,54,22) | 0.59* | 0.10 | 0.53 | 0.14 |
| Seed : Right dorsolateral prefrontal cortex |  |  |  |  |
| Superior Frontal (14,41,39) | 0.40* | 0.07 | 0.35 | 0.08 |
| Superior Temporal Pole (36,12,-21) | 0.05 | 0.05 | 0.05 | 0.05 |
| Temporal-Pole (38,12,-21) | 0.12 | 0.07 | 0.11 | 0.08 |

S.D.=standard deviation; PCC=posterior cingulate cortex

*p<0.05, Mann-Whitney U tests
