# Supplementary figures and images for: Associations of Bcl-2 rs956572 genotype groups in the structural covariance network in early-stage Alzheimer’s disease
Source: Alzheimers Res Ther. 2018 Feb 8;10:17. doi: 10.1186/s13195-018-0344-4 (PMC5806294; doi:10.1186/s13195-018-0344-4)

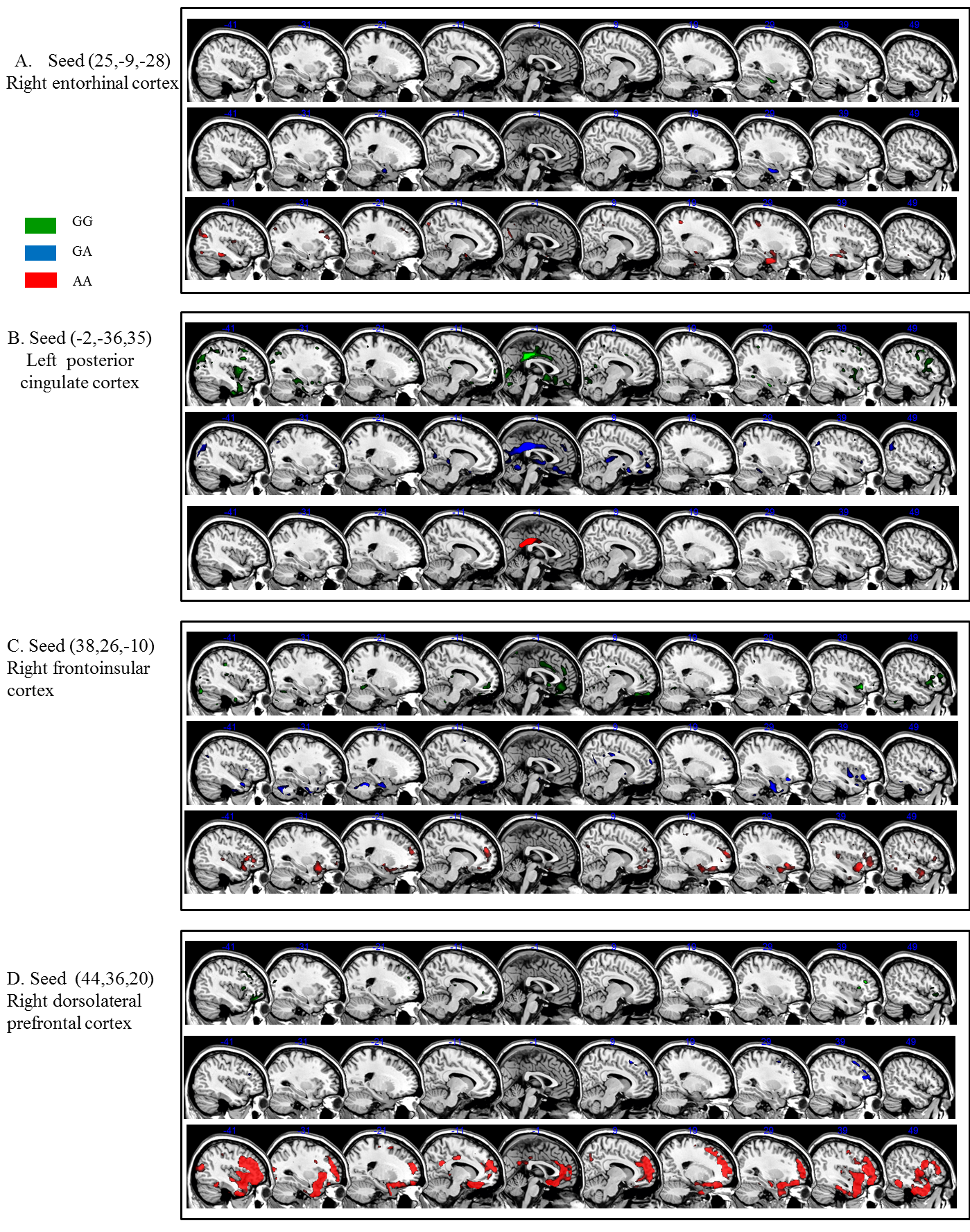

Supplement: Supplementary file 2 — Structural covariance networks from four seed regions in GG, GA, and A homozygotes. According to the case numbers, the actual T value in AA was 5.529, GA = 4.924, and GG = 6.4612. (BMP 7288 kb) [file 13195_2018_344_MOESM2_ESM.bmp]
